# PBX3 Promotes Tumor Growth and Angiogenesis via Activation of AT1R/VEGFR2 Pathway in Papillary Thyroid Carcinoma

**DOI:** 10.1155/2020/8954513

**Published:** 2020-01-25

**Authors:** Qin Chen, Wen-Ying Yu, Huan-Huan Zhang, Song-Zhao Zhang, Jie Fang, Fang Wu, Hua-Zhong Ying, Chen-Huan Yu

**Affiliations:** ^1^Department of Clinical Laboratory Medicine, Second Affiliated Hospital, College of Medicine, Zhejiang University, Hangzhou 310009, China; ^2^Key Laboratory of Experimental Animal and Safety Evaluation, Zhejiang Academy of Medical Sciences, Hangzhou 310013, China

## Abstract

PBX3 (Pre-B-cell leukemia homeobox 3) had been considered to be a multifunctional oncogene which involved in tumor growth, invasion, and metastasis in leukemia and some solid tumors. However, the contribution of PBX3 to papillary thyroid carcinoma (PTC) remains unclear. In this study, we found that PBX3 expression was significantly upregulated in PTC tissues compared to adjacent normal tissues, and high levels of PBX3 were correlated with tumor size, lymphatic metastasis, TMN stage, and poor prognosis of PTC patients. Overexpression of PBX3 in PTC cell lines promoted cell proliferation. Consistently, knockdown of PBX3 by shRNA induced cell cycle arrest at G0/G1 phase, and inhibited angiogenesis and tumor growth *in vitro* and *in vivo*. Furthermore, PBX3 promoted PTC cell proliferation and angiogenesis through activation of AT1R/VEGFR2 pathway while overexpression of AT1R and treatment with VEGFA reversed PBX3-shRNA-induced decreased phosphorylation of VEGFR2 and its downstream (ERK1/2, AKT and Src). It demonstrated that PBX3 could be used as a potential prognostic biomarker and therapeutic target for PTC.

## 1. Introduction

Thyroid cancer is one of the common cancers of the endocrine system. With the changing dietary habits in modern society, the incidence of this disease has increased in Asian countries, especially in coastal areas of China [1]. The most common type is papillary thyroid carcinoma (PTC), which accounts for approximately 85% of all primary thyroid malignancies. In most cases, well-differentiated PTC has an excellent prognosis. However, some patients die due to local recurrence, and distant metastasis [2]. The poor prognosis of PTC largely stems from insufficient understanding of tumor growth [3, 4]. Therefore, understanding the molecular mechanisms underlying tumor progression and finding specific prognostic biomarkers are clinically important.

Pre-B-cell leukemia homeobox 3 (PBX3) is a dominant cofactor in the PBX family, which can increase DNA-binding/transcriptional activity of homeobox (HOX) proteins, and its aberrant overexpression is required for the induction and maintenance of resistant acute myeloid leukemia [5–9]. PBX3 expression is also upregulated in some solid cancers and acts as a critical transcriptional switch for hepatoma stem cells [10–12]. Overexpression of PBX3 induces epithelial-mesenchymal transition and promotes invasion and metastasis of gastric, and colorectal cancer cells [13–15]. Moreover, elevated expression of PBX3 is associated with indolent progression to castration-resistant prostate cancer [16, 17]. Targeting HOXA9/PBX3 interaction has been suggested as a new therapeutic strategy to treat leukemia, gastrointestinal cancer, and nonsmall cell lung cancer [5, 18, 19]. However, the biological function of PBX3 in PTC remains unclear. In this study, we detected PBX3 expression in tissues from PTC patients and highlighted its new role in PTC cell proliferation and progression.

## 2. Materials and Methods

### 2.1. Clinical Tissue Specimens and Immunohistochemistry

Sixty-three paraffin-embedded PTC specimens were obtained from Zhejiang Academy of Medical Sciences. The clinicopathological information was recorded, and most patients were followed up from 2005 to 2015 after diagnosis. None of the patients were treated with any preoperative therapies such as radiation, chemotherapy, or immunotherapy before diagnosis. Fresh resected tumor samples and matched adjacent normal tissues from 20 well-established primary PTC cases were frozen in liquid nitrogen immediately after surgery and kept at −80°C until use. All the histology and clinical stages of the patients were classified according to the criteria of UICC (Union for International Cancer Control). The protocols of this clinical research were approved by the ethics committee of Zhejiang Academy of Medical Sciences, and all the patients gave their written informed consent. The tumor paraffin-cut sections were deparaffinized in xylene; rehydrated in graded ethanol solution; permeabilized in 0.1% Triton X-100; incubated overnight at 4°C with PBX3 antibody; and then treated with secondary antibody for 30 min at room temperature. The intensity and extent of staining were analyzed by using Image-Pro Plus software as reported previously [14].

### 2.2. Quantitative Real-Time PCR Analysis

Total RNA from clinical samples or cultured cells was extracted using the TRIZOL reagent (Invitrogen, USA) and then reverse transcribed with the cDNA Synthesis Kit (Takara, Japan). Quantitative real-time PCR (qRT-PCR) was performed using Roche SYBR Green PCR kits and carried out using a Jena Easy Cycler 96PCRsystem (Analytik Jena, Germany). The primer sequences were as follows: PBX3 forward, 5′-CAAGTCGGAGCCAATGTG-3′ and reverse, 5′-ATGTAGCTCAGGGAAAAGTG-3′; and GAPDH forward, 5′-CTGGGCTACACTGAGCACC-3′ and reverse, 5′-AAGTGGTCGTTGAGGGCAATG-3′.

### 2.3. Western Blot Analysis

Total proteins from cells or tumor tissues were extracted in lysis buffer and quantified using the Bradford method. Approximately 15 mg of protein was separated by SDS-PAGE. Samples were transferred to polyvinylidene difluoride membranes and incubated overnight at 4°C with antibodies (Proteintech, China) against PBX3, angiotensin II type 1 receptor (AT1R), AT1R-associated protein (ATRAP), VEGFR2, pTyr1175-VEGFR2, pY416-Src, ERK1/2, pT202/Y204-ERK1/2, AKT, pS473-AKT, and GAPDH. After incubation with peroxidase-coupled secondary antibody for 1 h at room temperature, bound signals were visualized using ECL and detected by using chemiluminescence imaging system (Bio-Rad, USA). Relative protein levels were quantified using GAPDH as loading control.

### 2.4. Cell Culture and Transfection

The PTC cell lines (TPC-1 and SW579) were cultured in Dulbecco's Modified Eagle Medium (DMEM) (Gibco, USA) and supplemented with 10% fetal bovine serum. For knockdown of endogenous PBX3 expression, shRNA-PBX3 (5′-GAGATTGAAAGAATGGTGGGC-3′) was designed and cloned into lentiviral vector plenti6-U6as previously reported. On the other hand, PBX3 cDNA was cloned into the lentiviral shuttle vector pCDH-CMV-MCS-EFl-Puro(LV-PBX3) purchased from System Biosciences, USA. The vectors were generated in 293T cells by using Lipofectamine 2000 (Invitrogen, USA) according to the manufacturer's instructions. The viruses were harvested 48 h after transfection, and then TPC-1 and SW579 cells were, respectively, infected with 2 × 10^6^ recombinant lentivirus-transducing units to obtain stable PBX3 overexpression or silence cell lines for subsequent assays. PTC cells were pretreated with pharmacological inhibitors for 30 min or transfected with siRNA, LV-PBX3, or vector for 24 h. Cells were then incubated with serum-free medium for two days. The medium was collected as conditioned medium (CM). The levels of VEGF in the CM were determined by ELISA assay.

### 2.5. Cell Proliferation Analysis

The transfected cells were cultured for 24–72 h. Subsequently, the cells were incubated with 1 mg/ml MTT-containing medium for 4 h. DMSO (100 *μ*l) was added to solubilize formazan after MTT was removed. The absorbance of samples was detected at 490 nm by a microplate reader (MD, USA).

### 2.6. Cell Cycle Analysis

The cells were cultured into 6-well plates at 1 × 10^6^ cells/well for analysis of cell viability. To analyze the cell cycle, cells were prepared using a Cell Cycle Detection Kit (KeyGen) and analyzed with a flow cytometer (BD Biosciences, USA).

### 2.7. Wound Healing Assay

Approximately 1 × 10^5^ cells/well were seeded into 6-well plates. The wounds were subsequently created into the confluent cell monolayer using a 100 *μ*l pipette tip after the transfected cell confluence reached 90%. Subsequently, culture plates were incubated at 37°C, and the migration of the cells was observed at 24 h.

### 2.8. Transwell Assay

The transwell assay was performed using migration chambers (BD Biosciences, NJ, USA) according to the manufacturer's instructions [20]. Cells were suspended in serum-free medium and added to the upper chamber, which was coated with (invasion assay) or without (migration assay) the matrigel mix. Culture medium with 20% fetal bovine serum was placed into the lower chamber. After 48 h, cells on the bottom of the membrane were fixed with 10% formalin, stained with 0.5% crystal violet, and finally washed twice with PBS. The number of invaded or migrated cells was counted from five different fields with a microscope (Olympus, Japan).

### 2.9. In Vitro Angiogenesis Analysis

Human umbilical vein endothelial cells (HUVECs) were seeded at the density of 2 × 10^4^ cells/well on 96-well plates, which were pre-coated with 50 *μ*l Matrigel (Gibco, USA), and cultured in tumor conditional medium (TCM), which contained 50% DMEM and 50% CM. The tube formation was photographed after 12 h and quantified by counting the tube branch points [21].

### 2.10. Chick Chorioallantoic Membrane (CAM) Assay

Fertilized chicken eggs (from Zhejiang Academy of Agricultural Sciences) were incubated at 39°C in the 80% humidified atmosphere. On day 7, TCM from differently treated cells was mixed with matrigel and deposited in the center of the egg. CAM was collected on the fourth day for microscopic and photographic documentation. At least 10 viable embryos were tested for each treatment.

### 2.11. In Vivo Tumor Xenograft Model

Forty-eight female BALB/c nude mice (six weeks old) were purchased from Siper-BK Co. Ltd., Shanghai, China. TPC-1 or SW579 cells transfected with shRNA-PBX3 or control shRNA were subcutaneously injected into the oxter of mice at a density of 1 × 10^6^ cells/ml. Each group has eight mice. Tumor volume was measured every four days with an external caliper, and the formula for calculation is shown as follows: *V*(mm^3^) = length × width^2^/2. The mice were sacrificed on the 24th day to evaluate tumor growth.

### 2.12. Statistical Analysis

Statistical analyses were performed using SPSS 17.0 software. The experiments were repeated three times independently, and the results were shown as the mean ± standard deviation. The differences between two groups were analyzed by Student's *t*-tests, and the data among multiple groups were analyzed using one-way analyses of variance or nonparametric Kruskal–Wallis *H* test. Values of *P* < 0.05 indicated a statistically significant difference.

## 3. Results

### 3.1. PBX3 Expression Was Upregulated in PTC Tissues and Cell Lines

**Figure 1:**
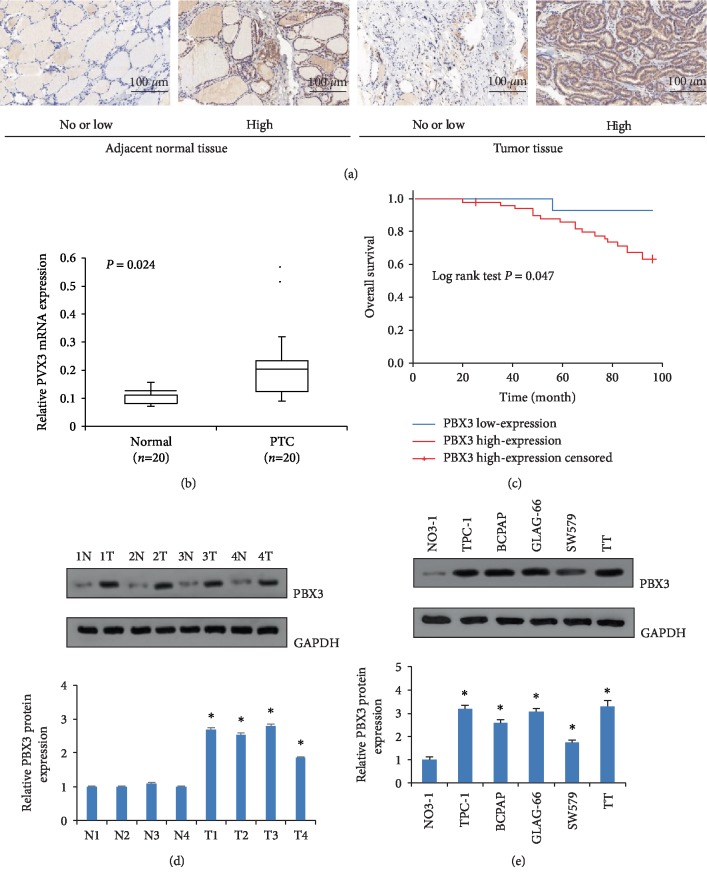
PBX3 is up-regulated in papillary thyroid carcinoma (PTC) and cancer cells. (a) Representative immunohistochemical staining of PBX3 protein in PTC tissues and adjacent normal tissues. (b) Expressions of PBX3 mRNA were significantly up-regulated in PTC (*n* = 20) compared with adjacent normal tissues (*n* = 20). (c) Patients with high PBX3 expression showed poor overall survival. (d) Expression of PBX3 protein in 4 representative paired samples of PTC tissues and adjacent normal tissues. (e) Up-regulation of PBX3 protein expression in PTC cells. ^∗^*P*0.05 versus normal control.

To investigate the effects of PBX3 in PTC progression, the profiles of PBX3 mRNA expression were also detected by qRT-PCR in 20 pairs of PTC samples. As shown in Figure.  1(b), PBX3 mRNA expression in PTC tissues was obviously higher than that in matched adjacent normal tissue. The results of western blot analysis further confirmed the upregulation of PBX3 protein in four randomly selected clinical samples (Figure 1(d)). Furthermore, PBX3 protein expressions were found to be two fold higher in five well-known PTC cell lines (TPC-1, BCPAP, GLAG-66, SW579, and TT) than that in the human normal thyroid cell line NO3-1 (Figure 1(e)). Given the growth capacity and tumorigenicity *in vivo*, TPC-1and SW579 cells were finally selected for further assay. These results demonstrated that PBX3 may contribute to the tumorigenesis of PTC.

To further explore the pathophysiological characteristics of PBX3 in PTC, the correlation between PBX3 expression and clinical features of PTC patients was analyzed by immunohistochemistry staining (Figure 1(a)). As shown in Table 1, high PBX3 expression was significantly associated with tumor size, lymphatic metastasis, and TMN stage (all *P* < 0.05). Furthermore, Kaplan–Meier analysis (Figure 1(c)) indicated that the PTC patients with high PBX3 expression had much poorer overall survival (*P* < 0.05). The results of multivariate Cox analysis showed that hazard ratio of PBX3 was 5.96 (95% confident interval: 0.80–44.65; *P* < 0.05), indicating that it could act as an independent prognostic factor in PTC patients.

### 3.2. PBX3 Promoted PTC Cell Proliferation, Migration, and Invasion

**Figure 2:**
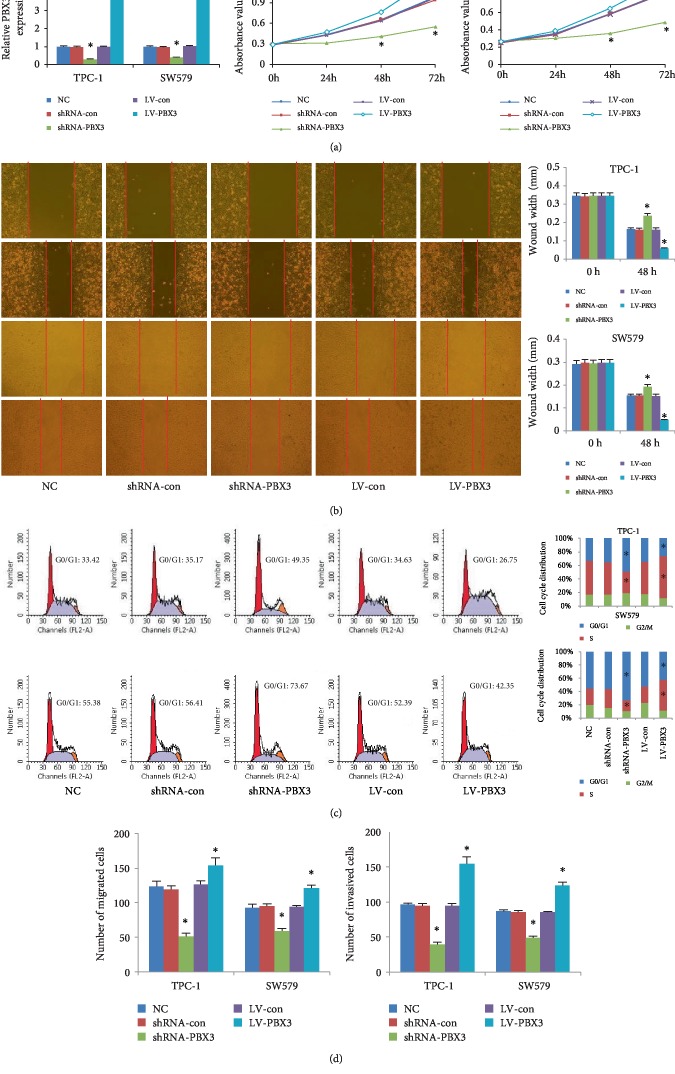
Effects of PBX3 on PTC cell proliferation, migration, and invasion. (a) The growth rates of PBX3-overexpressing or HPIP-knockdown PTC cells (TPC-1 and SW579). (b) Cell migration was determined over 48 h by wound healing assay. (c) The cell cycle was assessed by flow cytometry analysis. (d) Cell migration was assessed by transwell assays and cell invasion by the matrigel invasion chamber. All values are shown as mean ± SD (*n* = 3). ^∗^*P*0.05 versus control group.

To investigate the biological role of PBX3 in PTC cells, TPC-1and SW579 cells were stably infected with lentiviral vector expressing short hairpin RNA, which was designed to specifically knock down PBX3 expression. As shown in Figure 2, cell proliferation rates of PBX3-knockdown cells were significantly decreased compared with PTC cells infected with control shRNA. Knockdown of PBX3 also significantly inhibited cell migration and invasion by wound healing and transwell assays. On the other hand, PBX3 expression was also up-regulated over fivefold by transfecting lentivirus-transducing units into the cells. Moreover, PBX3 overexpression significantly promoted PTC cell proliferation, migration, and invasion.

### 3.3. PBX3 Accelerated G0/G1 Transitions in PTC Cells

Compared with control cells, overexpression of PBX3 in TPC-1 and SW579 cells significantly reduced the proportion of cells in the G0/G1 phase, but increased the proportion of cells in the S phase (Figure 2(c)). By contrast, the proportions of cells in the G0/G1 phase were significantly elevated after transfection with shRNA-PBX3. However, the proportions of cells in the S phase were decreased. These data suggest that PBX3 accelerated G0/G1 transitions in PTC cells.

### 3.4. PBX3 Promoted Cell Proliferation via Activating the AT1R/VEGFR2 Pathway

**Figure 3:**
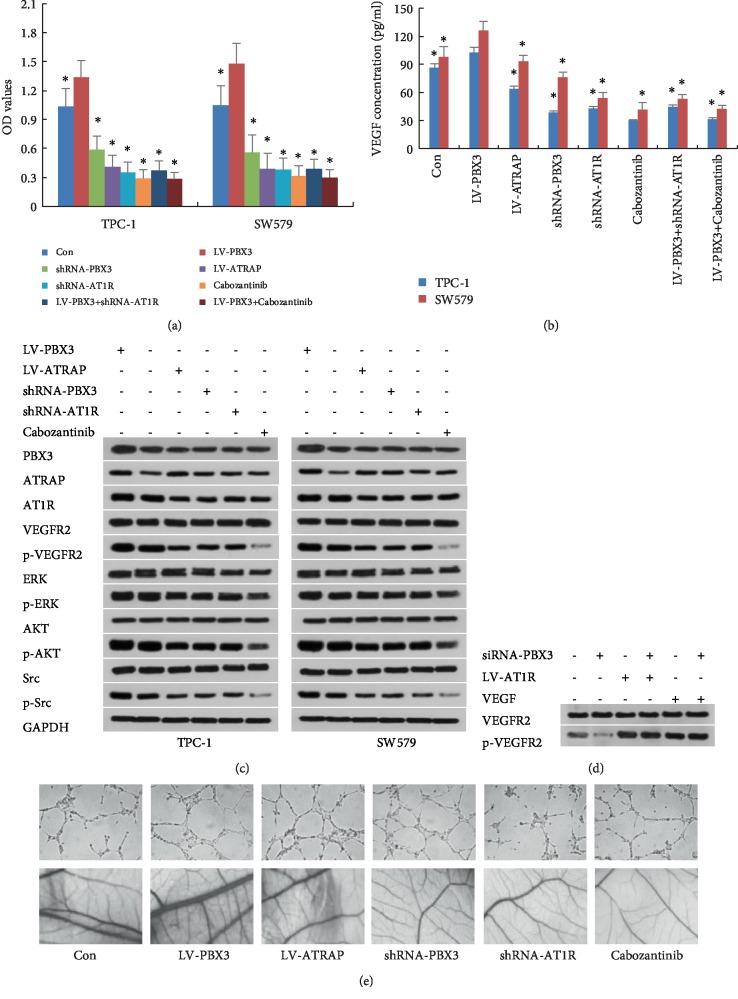
PBX3 promoted PTC cell proliferation and angiogenesis via activation of AT1R/VEGFR2 pathway. (a) Overexpression of ATRAP, knockdown of AT1R or cabozantinib treatment inhibited PTC cell proliferation, (b) inhibited VEGF production in cell culture, and (c) induced downregulation of VEGFR2 and its downstream (p-ERK1/2, p-AKT and p-Src). (d) AT1R overexpression and VEGFA administration rescued shRNA-PBX3-inhibited phosphorylation of VEGFR2. (e) The tube branch points of HUVECs (magnification, ×10) and angiogenesis of chick chorioallantoic membrane (magnification, ×10) induced by tumor conditioned medium treated with LV-PBX3, shRNA-PBX3, LV-ATRAP, shRNA-AT1R or cabozantinib. All values are shown as mean ± SD. ^∗^*P*0.05 versus control group.

The results in Figure 3 show that overexpression of PBX3 in TPC-1 and SW579 cells promoted cell proliferation, significantly upregulated the levels of AT1R but downregulated ATRAP, and increased the phosphorylation of VEGFR-2 and its downstream including ERK1/2, AKT, and Src compared with control cells (*P* < 0.05). By contrast, overexpression of ATRAP, silencing of AT1R, or treatment with VEGFR2 specificity inhibitor cabozantinib significantly reversed the PBX3-overexpression-induced proliferative effects in PTC cells and suppressed the levels of p-VEGFR-2, p-ERK1/2, p-AKT, and p-Src compared with the PBX3-overexpressed cells (*P* < 0.05). Furthermore, overexpression of AT1R or treatment with VEGFA rescued the decreased phosphorylation of VEGFR2 and VEGF production induced by inhibition of PBX3 shRNA. These results suggested that activation of AT1R/VEGFR2 pathway was responsible for PBX3 regulation of PTC cell proliferation.

### 3.5. Knockdown of PBX3 Inhibited Tumor Growth and Angiogenesis

**Figure 4:**
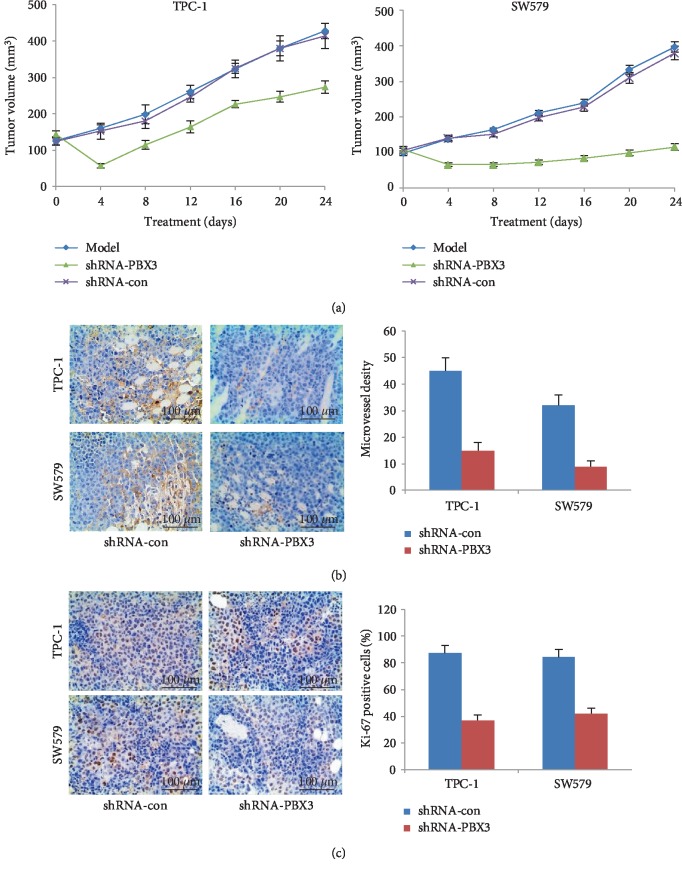
Knockdown of PBX3 suppressed PTC cell growth in nude mice. (a) HCT-8 cells stably infected with HPIP shRNA or control shRNA or parental HCT-8 cells were injected into nude mice, Tumor sizes were measured at the indicated times. Immunoblot analysis of (b) CD31, and (c) Ki-67 in tumor tissues. All values are shown as mean ± SD (*n* = 5). ^∗^*P*0.05 versus corresponding control shRNA.

PTC cells were transplanted subcutaneously into nude mice to investigate the effects of PBX3 on tumorigenic potential *in vivo*. As shown in Figure 4, the tumor volumes in mice injected with shRNA-PBX3-transfected TPC-1 and SW579 cells were much smaller than those in the control groups. Furthermore, knockdown of PBX3 made a significant decrease in endothelial vascular marker CD31 and cell proliferation marker Ki-67 expression in tumor tissues (Figures 4(b) and 4(c)). The results of tube formation analysis also showed that TCM with highest levels of VEGF in PBX3-overexpressed group enhanced HUVEC tube formation and CAM angiogenesis, whereas TCM in PBX3-knockdown group had lower levels of VEGF and obvious inhibition on HUVEC tube formation and CAM angiogenesis (Figures 3(c) and 3(d)). These results demonstrated that knockdown of PBX3 inhibited angiogenesis in PTC cells.

## 4. Discussion

Although significant technological progress has been made for the early diagnosis and prognosis of PTC, most screening techniques such as biopsy, ultrasound scan, and low-dose spiral CT scans are associated with some risk of overdiagnosis and false positives [2]. The mechanisms underlying PTC development, progression, and recurrence remain largely unknown. Therefore, identification of preventive strategies and therapeutic targets is urgent for PTC patients. In this study, the clinical significance and tumorigenesis of the novel cancer-related transcription fact or PBX3 were investigated for PTC.

Recent studies demonstrated that PBX3 acts as an oncogenic gene and participates in tumorigenesis, progression, and metastasis in many human malignancies including leukemia, multiple myeloma, gastrointestinal cancer, and prostate cancer [19]. It belongs to the TALE/PBXHOX family with a highly conserved homologous domain, which binds to the sequence 5′-ATCAATCAA-3′ by interacting with other homologous proteins (such asMEIS1 and HOXA9), resulting in transcription activation or target gene silencing [7, 9, 10]. Many reports demonstrated that aberrant expression of PBX3 was closely correlated with survival and tumor growth in patients with gastric and colorectal cancer [14–16]. Perturbing the PBX3 interaction with HOX or knockdown PBX3 induced apoptosis and inhibited epithelial-to-mesenchymal transition of tumor cells [9, 10]. Therefore, PBX3 could be a potential clinical prognostic biomarker and therapeutic target for gastrointestinal cancer. In this study, the expression of PBX3in PTC tumor tissues was significantly higher than that in adjacent normal tissues. PBX3 expression in PTC cells was also markedly increased compared with normal thyroid cells. Notably, the upregulated PBX3 expression was associated with tumor size, lymphatic metastasis, and TMN stage (all *P* < 0.05) as well as overall survival time of PTC patients. Kaplan–Meier and multivariate Cox regression analyses indicated PBX3 as an independent prognostic factor for PTC patients (hazard ratio = 5.96, 95% confident interval: 0.80–44.65, *P* < 0.05). These results indicated that PBX3 expression in tumor tissues could reflect the extent of malignancy and prognosis of PTC in part and be used as a potential clinical biomarker for evaluating PTC prognosis.

To explore the potential oncogenic function of PBX3 in PTC, two PTC cell lines TPC-1 and SW579 with high PBX3 expression and stable growth *in vivo* were transfected with shRNA-PBX3 or LV-PBX3. Overexpression of PBX3 accelerated PTC cell proliferation, migration, and invasion, but PBX3 knockdown inhibited these malignant behaviors. To further investigate the effect of PBX3 on PTC proliferation, cell cycle distribution was performed by flow cytometry analysis. The results of flow cytometry showed that cell proportion of the G0/G1 phase in the shRNA-PBX3 group was significantly increased compared with negative control, which was associated with significantly decreased cell percentage in *S* phase. Conversely, overexpression of PBX3 in TCP-1 and SW579 cells induced significantly decreased cell proportions of the G0/G1 phase, but significantly increased cell proportions of the *S* phase. Studies have demonstrated that dysregulation of the cell cycle is a remarkable characteristic of cancer cells. Transition from G1 to *S* phase requires the activation of cyclin D1 and A. In this study, we also found that the expressions of cyclin D1 and A were significantly increased in PBX3-overexpressed PTC cells compared with the negative group. These results demonstrated that knockdown of PBX3could induce cell cycle arrest at G0/G1 phase.

Angiogenesis is a complex multi-step process that includes destabilization of established vessels; proliferation, migration, and tube formation of endothelial cells; and remodeling of vascular basement membrane, leading to the formation of cancer [22]. Compelling evidence demonstrated that tumor cells secrete various cellular materials, such as cytokines, kinases, mRNAs, miRNAs, and exosomes, which have the potential to regulate normal cellular physiology and initiate robust invasion and metastasis via angiogenesis, immunosuppression and the epithelial-mesenchymal transition in the tumor micro-/macro-environment [23–25]. In this study, the tube branch points of HUVECs were significantly reduced under the TCM from the PBX3-knockdown cells compared with the control group; conversely, TCM from the PBX3-overexpressed cells could promote tube formation of HUVECs. Moreover, treatment with shRNA-PBX3 could significantly inhibit tumor growth as well as downregulate the expression of the microvessel biomarker CD31 in xenograft tumor tissues. It indicated that silencing of PBX3 inhibited the angiogenesis in PTC.

Emerging new researches had demonstrated that ATRAP directly binds to the C-terminus of AT1R, acts as a negative regulator of VEGFR2/angiotensin II (Ang II)-mediated signaling pathway by regulating ATIR internalization, and then induces a decrease in cell proliferation and Ang II (or VEGFA)-stimulated transcriptional activities, while PBX3 can form heteromeric complexes with ATRAP protein and inhibits ATRAP-caused the enhancement of AT1R internalization [26–28]. Our results showed that overexpression of PBX3 in PTC cells promote cell proliferation, increased VEGF production but decreased ATRAP expression, while knockdown of PBX3 led to diametrically opposite results. Previous report had shown that PBX3 induced migration and invasion of colorectal cancer cells partially through activation of ERK pathway [13]. However, in our study, we found that the decreased ATRAP expression in PTC cells caused the significant upregulation of AT1R, increased the phosphorylation of VEGFR2 and its downstream including ERK1/2, AKT, and Src; the increased VEGF production also promoted the excessive activation of VEGFR2 signaling pathway. Silencing AT1R expression or treatment with VEGFR2 inhibitor cabozantinib not only suppressed PTC cell proliferation, but also inhibited HUVEC tube formation as well as angiogenesis in CAM model. Moreover, overexpression of AT1R or administration with VEGFA rescued the decreased phosphorylation of VEGFR2 and VEGF production induced by inhibition of PBX3 shRNA. Thus, our findings demonstrated that activation of AT1R/VEGFR2 signaling pathway (as the upstream of ERK) was critical for PBX3-enhanced PTC cell proliferation and angiogenesis.

In conclusion, our results demonstrated that PBX3 was upregulated in human PTC tissues and associated with tumor size, lymphatic metastasis, and TMN stage. Inhibition of PBX3 induced apoptosis of PTC cells and suppressed angiogenesis via AT1R/VEGFR2 signaling pathway *in vitro* and *in vivo*. Thus, PBX3 may be a potential prognostic and therapeutic target for PTC treatment.

## Figures and Tables

**Table 1:** Correlation of PBX3 expression with clinicopathologic characteristics in PTC patients.

Clinicopathologic characteristics	*n*	PBX3 expression (%)		*χ* ^2^ value	*P* value
		Low expression	High expression		
Total	63	14	49		
Gender				0.001	0.971
Male	20	4 (20.0)	16 (80.0)		
Female	43	10 (23.3)	33 (76.7)		
Age (years)				0.114	0.736
≤50	34	7 (20.6)	27 (79.4)		
>50	29	7 (24.1)	22 (75.9)		
Tumor size (cm)				3.375	0.066
≤1	27	9 (33.3)	18 (66.7)		
>1	36	5 (13.9)	31 (86.1)		
Histopathological grade				0.198	0.657
G_1_ + G_2_	37	7 (18.9)	30 (81.1)		
G_3_	26	7 (26.9)	19 (73.1)		
Lymphatic metastasis				4.673	0.031
Absent	29	10 (34.5)	19 (65.5)		
Present	34	4 (11.8)	30 (88.2)		
TMN stage				8.490	0.004
T_1_ + T_2_	28	11 (39.3)	17 (60.7)		
T_3_ + T_4_	35	3 (8.6)	32 (91.4)		

## Data Availability

The data used to support the findings of this study are included within the article.
